# Public knowledge and willingness in the use of public access defibrillation of Hubei Province in China: A cross-sectional study

**DOI:** 10.1097/MD.0000000000036928

**Published:** 2024-01-19

**Authors:** Kaiqi Chen, Quan Yuan, Qianwen Zeng, Mengwan Liu, Cuihuan Hu

**Affiliations:** a School of Nursing, Tongji Medical College, Huazhong University of Science and Technology, Wuhan, China

**Keywords:** automated external defibrillator, awareness, public access defibrillation (PAD), resuscitation, survey

## Abstract

To understand the current status of public knowledge of automated external defibrillator (AED) and their willingness to use public AED in Hubei Province, along with the influencing factors. A self-designed questionnaire was used for convenience sampling of the public in Hubei Province. The questionnaire consists of three parts: basic information, AED knowledge questions, and willingness to use public AED and influencing factors. Data was collected between May 2022 and March 2023. A total of 1561 valid questionnaires were collected from 1602 distributed. In the study conducted in Hubei Province, it was found that 875 respondents (56.05%) had knowledge of automated external defibrillator, and they achieved an average score of 39.27 ± 29.17. The pass rate for the survey was 28.11%. Several factors were identified as significant influencing factors, including gender, age, education level, occupation related to medicine, residential location in the past three years, family members with cardiovascular disease, marital status, residential population density, whether there are family members over 65 years old, and participation in AED-related training (*P* < .05).Furthermore, 692 respondents (72.99%) expressed their willingness to cardiopulmonary resuscitation for someone experiencing cardiac arrest. On the other hand, 686 respondents (43.95%) had no knowledge of AED. Among those who were not willing to perform defibrillation, the highest percentages cited “fear of incorrect use” (129, 31.2%) and “fear of harming the patient” (121, 29.3%) as their reasons. The study also found statistically significant differences in the willingness to use public AED based on participation in training, education level, residential location, family members with cardiovascular disease, population density, and the presence of elderly family members aged 65 or over (*P* < .05). In conclusion, the study highlights the general lack of public knowledge regarding AED in Hubei Province. However, there is a strong willingness among respondents to provide help during cardiac arrest situations. To improve the chances of survival for cardiac arrest patients, it is crucial to strengthen public AED training programs.

## 1. Introduction

According to the “Chinese Cardiovascular Health and Disease Report” published by the National Health Commission, sudden cardiac arrest (SCA) affects approximately 544,000 people in China each year, with 75% of cases occurring outside of the hospital setting, such as in homes or public places.^[1]^ Unfortunately, many out-of-hospital cardiac arrest (OHCA) patients suffer permanent disability or death due to the lack of timely assistance or improper rescue efforts, underscoring the importance of prompt and accurate on-site emergency treatment over hospital treatment. Statistics have shown that 85% to 90% of OHCA patients experience ventricular fibrillation (VF) at the onset, and defibrillation is the fastest and most effective way to terminate VF. However, the effectiveness of defibrillation is time-sensitive, as every minute of delay in defibrillation reduces the survival rate of OHCA patients by 7% to 10%.^[2]^ If bystanders can use automatic external defibrillators (AEDs) to rescue patients in a timely manner, the success rate of rescuing OHCA patients would significantly increase. Reports indicate that the survival rate of patients with shockable rhythms is 38%, while the survival rate of patients with non-shockable rhythms is 9%.^[3]^ Recognizing the importance of AED usage, the American Heart Association (AHA) passed legislation in 1995 promoting public access defibrillation (PAD), which encourages the use of AED by the general public in public places. However, research shows that different countries have varying degrees of PAD implementation, with less than 3% of patients receiving defibrillation before the arrival of emergency medical services (EMS), and China having a lower implementation rate of less than 1%.^[4,5]^ The knowledge and willingness of the general public to perform cardiopulmonary resuscitation (CPR) and defibrillation in a timely manner are crucial factors that affect the effectiveness of first aid before professional medical personnel intervene. Therefore, this study aims to investigate the knowledge and willingness of AED use among the general public in Hubei Province, analyze the factors that influence AED knowledge and willingness, and provide scientific guidance to increase awareness of AED usage and improve the implementation rates of PAD treatments in the public. By understanding these factors, appropriate measures can be taken to enhance public engagement and improve the overall response to cardiac emergencies.

## 2. Methods

### 2.1. Study setting and data collection

A convenient sampling method was employed to select participants from the general population in various cities of Hubei Province, including Wuhan, Shiyan, Xiangyang, Xiaogan, Xianning, between May 2022 and March 2023. The study included individuals aged 15 years or older who had the ability to read or receive assistance in understanding the survey questionnaire, and were capable of independently completing the questionnaire. A total of 1602 questionnaires were distributed, and 1561 valid questionnaires were collected, resulting in a response rate of 97.4%. Among the respondents, 621 were males (39.8%) and 940 were females (60.2%). Prior to participating in the study, all participants were provided with information about the questionnaire's content, gave their informed consent, and willingly took part in the research.

### 2.2. Measures

Referring to domestic and foreign literature and based on the “2020 American Heart Association (AHA) Guidelines for Cardiopulmonary Resuscitation and Emergency Cardiovascular Care.”^[6]^ The research team developed and modified the content of the questionnaire after conducting a literature review and discussions. The questionnaire consisted of two dimensions: AED-related knowledge (10 items) and AED rescue usage attitudes (three items), resulting in a total of 13 items. To ensure the questionnaire's reliability and validity, a pre-experimental investigation was conducted with 165 participants from Hubei Province. The Cronbach’s α coefficient for the questionnaire was calculated to be 0.778, indicating good internal consistency. The validity of each question ranged from 0.504 to 0.774, further confirming the questionnaire's reliability. The questionnaire included three parts: general information: gender, age, ethnicity, education, whether the profession is related to medicine, place of residence in the past three years, whether there are family members living with cardiovascular disease, marital status, population density of residence, whether there are family members aged 65 and above living together, whether they have participated in CPR and defibrillation training, and religious beliefs; theoretical knowledge: including the steps for AED operation, applicable situations for AED usage, placement location for AED electrode pads, procedure for analyzing heart rate and giving a discharge, and other items; attitudes toward AED rescue usage: including obstacles, promoters, and factors affecting rescue implementation. The study received approval from the Medical Ethics Committee of Tongji Medical College, Huazhong University of Science and Technology.

### 2.3. Research team

This research team includes 6 graduate students and 12 experts in the field of CPR. Experts are included in the standard: First, Engaged in nursing (including clinical nursing or nursing education) or emergency medicine or emergency education in colleges or universities for > 10 years; second. Have a deep understanding of social first aid-related knowledge. Therefore, this study includes 12 experts, including doctors and nurses working in the emergency department of the hospital, emergency teachers of the Medical College, and first aid experts of the Red Cross, including 10 women and 2 men; 35 to 55 years old.

### 2.4. Data analysis

Excel 2019 software was used to establish a database for the data, and SPSS 27 was used for statistical analysis. Descriptive statistics, *t*-test, chi-square test, one-way ANOVA, and Binary logistic regression analysis were mainly used. Data was presented as mean ± standard deviation for quantitative variables, and rate for qualitative variables.

## 3. Results

### 3.1. Basic characteristics of study participants

In this study, a total of 1561 participants from Hubei Province were included. Among them, 621 participants (39.8%) were males, and 940 participants (60.2%) were females. The participants were selected from various cities in Hubei Province using a convenient sampling method. The study found that the largest proportion of participants who had knowledge about AED obtained the information through mobile phones, with 338 participants (38.7%) reporting this as their source. Lectures were the second most common source, with 264 participants (30.2%) acquiring knowledge through this medium. Computers were the source for 99 participants (11.3%), television for 118 participants (13.5%), and books and newspapers for 55 participants (6.3%). Regarding AED training, 307 participants (19.7%) reported having received training, while 1254 participants (80.3%) had not received any training. This indicates that a significant proportion of the general population in Hubei Province has not received formal AED training. In terms of residential areas, 1072 participants (68.7%) lived in urban areas, 309 participants (19.8%) lived in a town or suburb, and 180 participants (11.5%) lived in rural areas. Further details and characteristics of different participant groups can be found in Table 1 of the study.

**Table 1:** Respondents' demographic characteristics.

Investigation content	Total (*n*)	Percentage (%)
Gender		
Male	621	39.8
Female	940	60.2
Age		
Under 16 yr old	11	0.7
16 to 25 yr old	554	35.5
26 to 30 yr old	177	11.3
31 to 40 yr old	336	21.5
41 to 50 yr old	374	24
51 to 60 yr old	91	5.8
Over 60 yr old	18	1.2
Education		
Junior high school or below	192	12.3
High school or technical secondary school	364	23.3
Undergraduate or junior college	864	55.3
Postgraduate or above	71	4.5
Other	70	4.5
Whether the occupation is related to medicine (clinical)		
Yes	708	45.4
No	853	54.6
Place of residence (within the last three years)		
Urban	1072	68.7
A town or suburb	309	19.8
Rural area	180	11.5
Are there any patients with cardiovascular disease in the same family?		
Yes	756	48.4
No	805	51.6
Do you have cardiovascular disease?		
Yes	247	15.8
No	1314	84.2
The situation of marriage		
Be married	836	53.6
Spinster	643	41.2
Divorce or be bereaved of one's spouse	82	5.2
Population density of the place of residence		
High	390	25
Middle	812	52
Low	185	11.9
Unknown	174	11.1
Are there any family members over the age of 65 living together?		
Yes	722	46.3
No	839	53.7
Have you participated in the training of CPR or automatic external defibrillator?		
Yes	307	19.7
No	1254	80.3

CPR = cardiopulmonary resuscitation.

### 3.2. Investigation of awareness of AED among the survey participants

Regarding the level of understanding of AED, there are 127 people (8%) who have a high level of understanding, 134 people (8.5%) who have a good understanding, 304 people (19.5%) who have some understanding, 310 people (20%) who have little understanding, and 686 people (44%) who have no understanding at all.

And the occupation related to medicine and healthcare has a statistically significant effect on the understanding of AED (*P* ˂ .05), as shown in Table 2.

**Table 2:** Knowledge of AED in different occupations.

Knowledge of AED	Have no idea	A little understanding	Better understand	Know a lot about	Know very well
Number of people whose occupation is related to medicine (clinical)					
Yes	118(17.2%)	149(48.1%)	214(70.4%)	121(90.3%)	106(83.5%)
No	568(82.8%)	161(51.9%)	90(29.6%)	13(9.7%)	21(16.5%)
*χ* ^2^	480.848				
*P*	˂.001				

AED = automatic external defibrillator.

### 3.3. AED knowledge score

In the questionnaire, respondents were classified into one to five points based on their level of understanding of AED. Participants who scored one point, indicating complete unawareness, would skip to Question 25. Those scoring two to five points would answer questions related to AED knowledge. Among the individual questions, “When to use AED” had the highest correct rate, with 63.2% of respondents answering correctly. On the other hand, “Correct AED operation methods” had the lowest correct rate, with only 29% of respondents answering correctly. The number of individuals who answered each question correctly and the corresponding correct percentages are presented in Table 3 of the study. In terms of total knowledge scores, there were 10 AED-related questions, each worth 10 points, resulting in a total of 100 points. The passing score was set at 60. Among the participants, the highest proportion (20%) achieved a score of 50 points or above by answering half of the questions correctly. The second highest group (18.8%) received a score of 0, indicating a lack of knowledge on AED. Only 33 participants (4%) answered all questions correctly, demonstrating a high level of knowledge. The distribution of total AED knowledge scores is presented in Table 4. The study also analyzed the relationship between total knowledge scores and various demographic factors. The findings revealed that females scored higher than males, participants aged 16 to 25 scored higher than other age groups, individuals who had received AED training scored higher than those who had not, participants in healthcare-related professions or clinical medicine scored higher than those in non-related professions, residents in high population density urban areas scored higher than those in low-density areas, and individuals with a master's degree or higher scored higher than those with lower educational levels. Moreover, participants who lived with family members over the age of 65 also scored higher than those who did not live with family members over the age of 65. These results highlight the variations in AED knowledge levels among different demographic groups and suggest the influence of factors such as gender, age, education level, occupation, training, and living arrangements on AED knowledge scores.

**Table 3:** Scores for AED-related knowledge questions among survey participants.

AED-related knowledge questions	Number of correct answers	Percentage of correct answers (%)
1. When should AED be used?	553	63.2
2. What is the correct method for operating AED?	255	29.0
3. Where should AED electrode pads be placed?	306	35.0
4. What is the correct way to apply AED electrode pads?	554	63.0
5. What measures should rescuers take when AED analyzes heart rate?	298	34.0
6. What is the correct procedure for discharging AED?	310	35.0
7. Which of the following is incorrect about AED?	309	35.0
8. What should rescuers do after delivering a shock during CPR?	336	38.0
9. Is CPR necessary when AED is available?	511	58.0
10. Why is AED important for out-of-hospital cardiac arrest patients?	558	64.0

AED = automatic external defibrillator, CPR = cardiopulmonary resuscitation.

**Table 4:** Total score of AED knowledge among respondents.

Total AED score	The number of correct answers	Take up a proportion of (%)
0	165	18.8
10	78	9.0
20	98	11.2
30	4	0.5
40	113	13.0
50	171	20.0
60	83	9.0
70	80	9.0
90	50	5.5
100	33	4.0
Total	875	100.0

AED = automatic external defibrillator.

### 3.4. Comparison of the willingness to implement PAD among different demographic groups

Univariate analysis was conducted to examine the intention of the respondents to implement PAD. The respondents were grouped based on various factors, including gender, marital status, presence of cardiovascular disease patients in their relatives, population density, personal history of cardiovascular disease, presence of elderly people over 65 years old living with them, age, education level, participation in CPR training, place of residence (in the past three years), and participation in CPR training.

The results of the analysis revealed that several factors had a statistically significant influence on the intention to implement PAD (*P* < .05). These factors included the presence of cardiovascular disease patients in relatives, population density, presence of elderly people over 65 years old living with them, age, education level, participation in CPR training, place of residence (in the past three years), and participation in CPR training. Table 5 of the study presents the differences in the willingness to perform CPR among individuals with different characteristics. This table provides a comprehensive overview of the statistical significance and impact of various factors on the intention to implement PAD among the respondents. These findings suggest that factors such as personal and familial medical history, living environment, age, education, and previous CPR training play a significant role in influencing individuals' willingness to implement PAD. Understanding these factors can help guide efforts to promote and increase the adoption of PAD initiatives in the population.

**Table 5:** Univariate analysis of respondents' willingness to implement PAD and AED knowledge score.

Item	Willing to activate public access defibrillation for rescue	*χ* ^2^	*P* value
Have you participated in the training of CPR or automatic external defibrillator?		36.293	.001
Yes	235(34%)		
No	457(66%)		
Education		17.279	.002
Junior high school or below	63(9.1%)		
High school or technical secondary school	163(23.6%)		
Undergraduate or junior college	407(58.8%)		
Postgraduate or above	35(5.1%)		
Unknown	24(3.5%)		
Place of residence (within the last three years)		16.005	.002
Urban	489(70.7%)		
A town or suburb	135(19.5)		
Rural area	68(9.8%)		
Are there any patients with cardiovascular disease in the same family?		11.65	.006
Yes	232(22.4%)		
No	43(19.3%)		
Population density of the place of residence		14.52	.008
High	190(27.5%)		
Middle	369(53.3%)		
Low	81(11.7%)		
Unknown	52(7.5%)		
Are there any family members over the age of 65 living together?		12.807	.007
Yes	369(53.3%)		
No	323(46.7%)		
Whether the occupation is related to medicine (clinical)		4.315	.038
Yes	474(68.5%)		
No	218(31.5%)		
Age			
Under 16 yr old	5(0.7%)	28.298	.001
16 to 25 yr old	212(30.6%)		
26 to 30 yr old	103(14.9%)		
31 to 40 yr old	169(24.4%)		
41 to 50 yr old	156(22.5%)		
51 to 60 yr old	40(5.8%)		
Over 60 yr old	7(1.0%)		

AED = automatic external defibrillators, CPR = cardiopulmonary resuscitation, PAD = public access defibrillation.

### 3.5. Binary logistic regression analysis of influencing factors of defibrillation rescue intention

In this study, the dependent variable is whether they are willing to use defibrillator for rescue (yes/no). There are eight independent variables and categorical variables (Place of residence: 1 = city, 2 = town or suburb, 3 = countryside; Population density: 1 = high, 2 = medium, 3 = low; Participated in training: 1 = yes, 2 = no; Whether occupation is related to medicine: 1 = yes, 2 = no; Whether there are people over 65 years old living with them: 1 = yes, 2 = no; Whether there is cardiovascular disease in the family members living with:1 = yes, 2 = no; Education: 1 = junior high school and below, 2 = senior high school and technical secondary school, 3 = undergraduate, junior college, 4 = master degree and above, 5 = other; Age: 1 = under 16 years old, 2 = 16–25 years old, 3 = 26–30 years old, 4 = 31–40 years old, 5 = 41–50 years old, 6 = 51–60 years old, 7 = over 60 years old) that is, it is currently known that the factors influencing the willingness to use defibrillators for rescue may be age, education, place of residence, population density, whether they have participated in training, whether their occupation is related to medicine, whether there are older people over 65 years old living with them, and whether there are cardiovascular diseases in their family members living with them. Therefore, after collecting the data, the influencing factors of rescue willingness were studied by binary logistic regression.

First, multicollinearity is tested, which is similar to linear regression. In binary logistic regression, the correlation of independent variables should not be too high. If it is too high, multicollinearity will appear and affect the final estimation result. In binary logistic regression, the diagnosis method of linearity in linear regression can be used to determine whether there is multicollinearity between independent variables. It can be seen from the Table 6 that the variance inflation factor VIF values of the independent variables are all small, less than 5, indicating that there is no multicollinearity problem between explanatory variables.

**Table 6:** Multicollinearity analysis.

Model	Unnormalized coefficient		Standardization coefficient	*t*	Significance	Collinearity statistics	
	*B*	Standard error	Beta			Allowance	VIF
(Constant)	0.954	0.114		8.336	0		
Education	−-.031	0.017	−0.061	−1.82	0.069	0.857	1.166
Whether the occupation is related to medicine (clinical)	0.011	0.031	0.012	0.365	0.715	0.883	1.132
Place of residence	0.025	0.022	0.039	1.168	0.243	0.858	1.165
Patients with cardiovascular disease in the same family	0.024	0.029	0.027	0.825	0.41	0.899	1.112
Population density of the place of residence	0.037	0.016	0.072	2.248	0.025	0.936	1.068
Family members over the age of 65	0.1	0.028	0.113	3.517	0	0.943	1.06
Training	0.157	0.034	0.16	4.652	0	0.817	1.224
Age	−0.053	0.011	−0.16	−4.826	0	0.877	1.141

The single factor *P* < .05 was then carried out by binary logistic regression, and the fitting result of the model could be obtained at first. It could be seen from the Omnibus test of the model coefficient that the corresponding *P* value was less than .001, so it was definitely less than .05, indicating that the model was effective and the model fitted well.

The results in Table 7 show that population density is an influential factor of rescue intention, and the rescue intention of individuals with “medium” population density is 0.498 times that of individuals with “high” population density. The rescue willingness of individuals with “low” population density was 0.521 times that of individuals with “high” population density. The willingness to help was 0.52 times higher in individuals with a “don't know” population density than in those with a “high” population density. “Whether there is a family member over 65 years old living with him or her” is an influential factor of rescue willingness, and the willingness to rescue of individuals with “no family member over 65 years old living with him or her” is 0.584 times that of individuals with “family member over 65 years old living with him or her living with him or her.” Whether participated in the training or not was the influential factor of rescue willingness, and the individual who did not participate in the rescue willingness was 0.403 times that of the individual who had participated in the rescue willingness.

**Table 7:** Binary logistic regression of PAD implementation intention.

Variable in an equation	*B*	Standard error	Wald	Significance	Exp(*B*)
Age			19.951	0.003	
Age(1)	0.881	1.377	0.41	0.522	2.414
Age(2)	1.464	1.099	1.774	0.183	4.322
Age(3)	1.195	1.106	1.167	0.28	3.302
Age(4)	0.947	1.1	0.741	0.389	2.578
Age(5)	0.566	1.103	0.263	0.608	1.76
Age(6)	0.081	1.174	0.005	0.945	1.085
Education			10.202	0.037	
Education(1)	0.693	0.445	2.424	0.119	2
Education(2)	0.145	0.421	0.119	0.73	1.156
Education(3)	−0.082	0.389	0.044	0.834	0.922
Education(4)	0.395	0.483	0.668	0.414	1.485
Whether the occupation is related to medicine (clinical)(1)	−0.1	0.174	0.33	0.566	0.905
Place of residence			1.769	0.413	
Place of residence(1)	−0.156	0.246	0.403	0.525	0.855
Place of residence(2)	0.095	0.268	0.125	0.724	1.099
Patients with cardiovascular disease in the same family(1)	−0.105	0.165	0.405	0.524	0.9
Population density of the place of residence			6.976	0.073	
Population density of the place of residence(1)	−0.697	0.288	5.836	0.016	0.498
Population density of the place of residence(2)	−0.653	0.263	6.141	0.013	0.521
Population density of the place of residence(3)	−0.653	0.326	4.016	0.045	0.52
Family members over the age of 65(1)	−0.539	0.162	11.123	0.001	0.584
Training(1)	−0.984	0.223	19.561	0	0.374
Constant	−0.908	1.196	0.576	0.448	0.403

### 3.6. Hindrance factors, promotion factors, and rescue factors affecting PAD implementation by the public

When asked about their willingness to assist in a public emergency, participants were presented with a scenario: If someone were to suddenly collapse in front of them, showing no signs of responsiveness, breathing, or only gasping, would they step forward to assess the situation and utilize an automated external defibrillator to initiate rescue efforts? 948 of the public responded, 73% of the public are willing to start PAD program to help OHCA patients who fall on the ground, and 27% of the public are not willing to help. This shows that most of the public in China are willing to rescue, among which the most encouraging factors are “saving instinct” and “responsibility,” while the public who are not willing to rescue are more concerned about “fear that they cannot use correctly” and “fear of accidentally injuring patients.” The public believes that the factors that most affect their own rescue are “whether the environment is safe” and “public opinion pressure.” The factors affecting PAD implementation by the public are shown in Table 8.

**Table 8:** Hindering factors, promoting factors, and influencing factors of public rescue.

Item	Select number of people	Take up a proportion of
1. Obstacle factors affecting public rescue (choose not to save)		
A. Fear of no legal guarantee and fear of liability	66	16.00%
B. Worry that you won't be able to use it properly	129	31.20%
C. Fear of accidentally injuring patients	121	29.30%
D. Unwilling to take risks, will call 120 for help	88	21.30%
E. Other factors	9	2.20%
2. Promoting factors affecting public rescue (choose to save)		
A. The instinct to save people	482	38.90%
B. Where the responsibility lies	362	29.20%
C. I can use AED	227	18.30%
D. I have confidence in myself	157	12.70%
E. Other factors	12	1.00%
3. Factors affecting rescue (multiple choices)		
A. Personal values	262	18.80%
B. Whether the environment is safe	461	33.10%
C. Pressure from public opinion	339	24.30%
D. Personality traits	241	17.30%
E. I don't know	91	6.50%

AED = automatic external defibrillator.

## 4. Discussion

This study highlights the low level of knowledge about AED among the general public in Hubei province, while also indicating a positive attitude toward performing CPR. To address this issue, it is recommended to strengthen AED training, increase the availability of AED in Hubei province, and promote AED through positive social publicity. These efforts aim to enhance the public's ability to save the lives of OHCA patients. Among the survey participants who had received training, the majority obtained their knowledge through mobile phones, followed by attending courses or lectures. Individuals with medical or related backgrounds demonstrated a higher level of understanding of AED compared to those without such backgrounds, which may be attributed to training organized by hospitals or medical institutions. The study found that 63.2% of the public knew “when to use AED,” but only 29% answered correctly regarding the correct method of using AED. This indicates a lack of a government-led, professional training system, and management approach for AED. AED remain a term familiar primarily to the medical field rather than the general public. However, China does offer training courses on the use of AED. For instance, the “Red Cross Rescuer” training project carried out by the Red Cross includes relevant content related to CPR. Applicants voluntarily fill in the China Red Cross Rescuer Information Form, and if they meet the requirements, they are notified to participate in the Rescuer training. Those who pass the rescue assessment receive a certificate, and the “Red Cross Rescuer” certificate can exempt individuals from liability during emergency relief. The study findings also indicate that women scored higher than men, people aged 16 to 25 scored higher than other age groups, those who had received training scored higher than those who had not, individuals with medical or related backgrounds scored higher than those without, individuals living in areas with higher population densities scored higher, those living in cities scored higher than those in rural areas, individuals with graduate degrees scored higher than those with other educational levels, and those with family members over 65 years old scored higher than those without. These results suggest that younger individuals, those with medical and related backgrounds, those with higher education, and those with family members at risk of cardiac arrest have better knowledge of AED. Younger individuals tend to have a higher cognitive level and better access to knowledge through their active engagement in schools, society, and families. Therefore, training activities can be initiated by relevant institutions and family members to increase the likelihood of effective training. Similarly, family members at risk of cardiac arrest display a higher level of AED knowledge, indicating that AED promotion can be improved through disease prevention publicity and related chronic disease management approaches in hospitals and public health organizations. Enhancing AED knowledge can be achieved through training and improving the availability of AED through various means. The survey results indicate that a significant portion of the general public in Hubei province lacks knowledge about AED, with 44.0% of respondents having no knowledge of AED, and only 19.7% having received AED-related training. These findings underscore the importance of implementing educational initiatives and increasing awareness about AED to improve public preparedness in responding to cardiac emergencies. This is similar to the results of Nie Shaozhou et al.^[7]^ In the United States, one in four people can perform CPR, this shows that there are still shortcomings in the popularization of AED in our province, and also reflects the urgency of PAD plan. Data show that the public penetration rate of AED education is 79% in the United States, 74% in Australia, 64% in Canada,^[8]^ 61% in the United Kingdom,^[9]^ 31.1% in West Africa,^[10]^ whereas only 1% of the Chinese public qualified for AED training for OHCA.^[11]^ Therefore, to enhance knowledge and skills training, the Global Resuscitation Alliance proposes CPR and AED training in schools and communities to improve OHCA survival rates, as emphasized in the “Saving Lives of Children” declaration, which has substantial evidence that school-age children can play a critical role in improving OHCA survival rates.^[12]^ The 2018 consensus proposal for CPR training in China suggested that community volunteers and healthcare workers should receive basic first aid skills training through lectures, videos, and informational materials.^[13]^ In the 2021 National People's Congress, Shenzhen proposed the “Shenzhen Plan” for promoting AED use. While there are many AED available, often no one knows how to use them or feels confident enough to do so. Therefore, it is essential to ensure that the number of AED placements is close to certain countries' averages, guaranteeing that AED are easily accessible in the event of an OHCA. At the same time, it is critical to expand the diverse training pathways for individuals, families, hospitals, communities, and society. For example, in Denmark, most people aged 18 to 29 have received CPR training when obtaining their driver's licenses,^[14]^ while the British Heart Foundation's Heart Start Day offers relevant training courses to promote the availability of CPR and defibrillator training for the general public. Therefore, it is essential to enhance professional emergency training teams in order to comprehensively enhance the public's knowledge and skills in AED and CPR. This will ultimately increase the likelihood of successful prehospital emergency care.

A positive social propaganda of AED and knowledge dissemination can increase people's willingness to help in emergency situations. AHA guidelines emphasize the need for synchronous “implementation and training,” both focusing on knowledge and skills training and improving rescuers' behavioral intentions.^[15]^ In this study, further analysis of public willingness to perform PAD was conducted, revealing that 72.99% of the public is willing to implement PAD for emergency assistance. Several researchers expressed a strong desire for further training and learning in offline surveys, which this research group will provide through training activities. Studies have shown that people's personal psychology, including environmental factors, individual abilities, and the health of rescuers, can affect their willingness to help in emergencies. Confidence in their own abilities is the biggest factor hindering people from performing emergency care.^[16]^ Although training can provide the necessary skills, task-oriented learning methods may not effectively enhance a person's motivation to rescue. The factors influencing rescuer willingness were designed based on the “willingness-centered” bystander model theory,^[17]^ such as the planned behavior theory. An individual's willingness to help is influenced by their beliefs, which shape their personal attitudes toward rescue (positive or negative), their perception of social norms (how people view non-helping behavior and social pressure), and their perceived ability to predict the outcome of their actions (self-efficacy, personality traits). This study identifies the most significant factors influencing rescuer willingness as the safety of the environment and social pressure. Social pressure is found to have the greatest impact on rescue behavior, apart from the rescue environment.

In addition to training, which is an important factor, China can promote education and awareness about AED usage through various forms of mass media and online activities. Emphasizing the key points of AED usage and highlighting its importance in saving lives of OHCA patients can help overcome and reduce fear, thus increasing the public's willingness to help. It is also crucial to address the issue of rights and responsibilities during the AED usage process. Implementing comprehensive training programs and efficient propaganda systems based on modern information media can further enhance public knowledge and engagement with AED.

Furthermore, this study reveals that in areas with high population density, among individuals living with elderly persons aged 65 or older, and among family members with cardiovascular diseases, the public is more inclined to intervene and provide help. This trend may be attributed to the aging population and changing disease patterns in China, where cardiovascular diseases and accidental injuries have become leading causes of death. As the population ages, it becomes increasingly important for society to actively participate in learning first aid knowledge, such as CPR and defibrillation. Emphasizing the societal and moral standards of rescuers and their actions, along with shaping public opinion, can contribute to creating a positive cycle. Additionally, actively promoting government legislation, social response, and implementation across various sectors can further strengthen the overall response to cardiac emergencies. By fostering a collective effort, society can enhance its ability to respond effectively to such situations and establish a culture of proactive assistance. Therefore, members of the public cannot only use AED but also dare to use them and use them effectively while facing OHCA occurrences.^[18]^

This survey used a combination of online and offline questionnaires, and the quality of the questionnaire may vary, but the survey was conducted randomly and voluntarily, and the questionnaire results are trustworthy. The age groups with the highest percentage of responses were 16 to 25 and 41 to 50, with most respondents being high school or university students and middle-aged with sufficient social experience, respectively. They reflect the lack of AED training courses for emergency response systems and the promotion and training progress of AED among the general public in the future in response to the population aging trend.

## 5. Conclusion

In conclusion, the public in Hubei province generally lack awareness of AED-related knowledge but have a strong willingness to intervene and provide help to patients who need PAD. Therefore, efforts need to be made to increase the public's awareness and willingness to implement AED measures, including extending knowledge education from schools to communities, organizing advertising campaigns in newspapers, websites, and social media, and expanding the influence of the crowd through CPR training lectures, teaching videos, and social networks.

## Author contributions

**Data curation:** Cuihuan Hu.

**Formal analysis:** Qianwen Zeng.

**Funding acquisition:** Cuihuan Hu.

**Investigation:** Cuihuan Hu.

**Software:** Mengwan Liu.

**Writing—original draft:** Kaiqi Chen.

**Writing—review and editing:** Quan Yuan.
